# Prevention of Intraabdominal Adhesions by Local and Systemic Administration of Immunosuppressive Drugs

**DOI:** 10.5812/ircmj.14148

**Published:** 2013-12-05

**Authors:** Kemal Peker, Abdullah Inal, Ilyas Sayar, Murat Sahin, Huriye Gullu, Duriye Gul Inal, Arda Isik

**Affiliations:** 1 Department of General Surgery, Erzincan University, Erzincan, Turkey; 2 Department of Pathology, Erzincan University, Erzincan, Turkey; 3 Department of Anesthesiology & Reanimation, Erzincan University, Erzincan, Turkey

**Keywords:** Pharmaceutical Preparations, Immunosuppression, Peritoneum, General Surgery

## Abstract

**Background:**

Intraperitoneal adhesion formation is a serious postsurgical issue. Adhesions develop after damage to the peritoneum by surgery, irradiation, infection or trauma.

**Objectives:**

Using a rat model, we compared the effectiveness of systemic and intraperitoneally administered common immunosuppressive drugs for prevention of postoperative intraperitoneal adhesions.

**Materials and Methods:**

Peritoneal adhesions were induced in 98 female Wistar-Albino rats by cecal abrasion and peritoneal excision. Rats were randomly separated into seven groups, each containing fourteen rats, and the standard experimental model was applied to all of rats. 14 days later, rats were euthanized, intraperitoneal adhesions were scored and tissues were examined histologically using hematoxylin/eosin and Masson’s trichrome staining.

**Results:**

Throughout the investigation, no animal died during or after surgery. In all of experimental groups, decrease in fibrosis was statistically significant. Decrease in fibrosis was most prominently in intraperitoneal tacrolimus group (P = 0.000), and decrease was least in intraperitoneal cyclosporine group (P = 0.022). Vascular proliferation was significantly decreased in all experimental groups (P < 0.05) except for systemic tacrolimus group (P = 0.139). Most prominent reduction in vascular proliferation was in intraperitoneal tacrolimus group (P = 0.000).

**Conclusions:**

Administration of immunosuppressive drugs is effective for prevention of intraperitoneal adhesions.

## 1. Background

Postoperative peritoneal adhesions are often associated with any kind of abdominal surgery, and may lead to significant clinical, economic and legal consequences (1). Chronic pelvic pain, primary and secondary female infertility and intestinal obstructions are some of well-known serious postoperative problems which is associated with adhesion formation (2). Peritoneal adhesions cause more than 60% of all small bowel obstructions; which is probably the most severe consequence of postoperative adhesions (3). Pathophysiology of adhesion formation after abdomino-pelvic surgery appears to be involved with complex local and systemic inﬂammatory responses due to peritoneal injury. Various cytokines,chemokines and proteases which inﬂuence ﬁbrinogenesis, ﬁbrinolysis, angiogenesis and tissue remodeling after surgical injury may regulate these inﬂammatory responses (4). Peritoneal adhesions may be classified as congenital or acquired. Congenital adhesions are present from birth as an embryological anomaly in the development of the peritoneal cavity. Acquired adhesions may either be inflammatory or postsurgical. Inflammatory adhesions arise from intra-abdominal inflammatory processes. Postsurgical adhesions, which constitute the majority of the peritoneal adhesions, are consequence of injured tissue surfaces following incision, cauterization, suturing or other means of mesothelial trauma (5). Inflammatory response probably has a pivotal role in peritoneal adhesion formation through immune cells and mediators (6, 7). Peritoneal adhesions generally begin to be formed in the early postoperative period. Various agents against fibrinous adhesion formation have been tried with promise results in animal models (8). Along with these findings, observed incidence of postoperative adhesion-related intestinal obstruction after visceral organ transplantation has remained very low (9, 10). In a retrospective review of 4001 cases of orthotopic liver transplantation, 48 patients (1.2%) had postoperative bowel obstruction. Furthermore, obstruction was directly related to peritoneal adhesions only in 19 cases (0.5%) (11). On the other hand, still there are not sufficient experimental or clinical data for effect of immunosuppressive agents on adhesion formation.

## 2. Objectives

The purpose of this study was to investigate the inﬂuence of common immunosuppressive drugs on adhesion formation after abdominal surgery in an experimental rat model.

## 3. Materials and Methods

This experimental study was approved by the Ethics Committee on Animal Research of Ataturk University, Erzurum, Turkey with approval code of 4/47 in 26 April 2012. Study was conducted in conformity with instutional standards of Ataturk University. A total of 98 female Wistar-Albino rats weighing between 300 - 350 g were used for the study (n = 98; median weight = 324,0 g; mean weight (±SD) = 325, 66 ± 13,177 g) with expected sample power of 88%. All animals were housed at standard laboratory conditions (temperature 21 ± 2 ºC 12h light/12h dark, relative humidity 50%) and fed with standard rodent chow and water ad libitum. Animals were fasted for 8 hours prior to procedure and fed with standard diet 8 hours after surgery, postoperatively. Animals were anesthetized by intramuscular administration of 75 mg/kg ketamine (Ketalar® 50 mg/mL, Eczacıbasi, Istanbul, Turkey) and 5 mg/kg xylazine (Rompun® 23.32 mg/mL, Bayer, Istanbul, Turkey). Animals were placed in supine position, abdomens were washed with chlorhexidine solution, shaved and surgical fields were prepared with 10% povidone-iodine solution. After sterile draping, adhesion models were created in aseptic conditions. Laparotomy was made through 3 cm midline incision. Cecum was exteriorized and abraded with a sterile gauze pad until punctate hemorrhage was seen. Right-sided parietal peritoneum was excised.

### 3.1. Experimental Groups

Rats were randomly separated into seven groups each containing 14 rats, and standard experimental model was applied to all of rats.

#### 3.1.1. Control Group- Group1 (G1)

Adhesion model was performed. 2 ml of 0.9% saline solution was administered in peritoneal cavity and then incision was closed.

#### 3.1.2. Systemic Mycophenolate Mofetil Group- Group2 (G2)

10 mg/kg Mycophenolate mofetil was administered orally by an oral gavage tube under general anesthesia one hour before adhesion model was created.

#### 3.1.3. Intraperitoneal Mycophenolate Mofetil Group-Group3 (G3)

Adhesion model was performed. 10 mg / kg Mycophenolate mofetil in 1 mL saline was administered in peritoneal cavity and then incision was closed.

#### 3.1.4. Systemic Tacrolimus Group-Group4 (G4)

0.30 mg/kg tacrolimus was administered orally by an oral gavage tube under general anesthesia one hour before adhesion model was created. 

#### 3.1.5. Intraperitoneal Tacrolimus Group-Group5 (G5)

Adhesion model was performed. 0.10 mg/kg tacrolimus in 1 mL saline was administered in peritoneal cavity and then incision was closed.

#### 3.1.6. Systemic Cyclosporine Group-Group6 (G6)

10 mg/kg cycosporine was administered orally by an oral gavage tube under general anesthesia one hour before adhesion model was created.

#### 3.1.7. Intraperitoneal Cyclosporine Group-Group7 (G7)

Adhesion model was performed. 1 mg/kg cyclosporine in 1 mL saline was administered in peritoneal cavity and then incision was closed.

### 3.2. Parameters

#### 3.2.1. Adhesion Score

**Figure 1.**
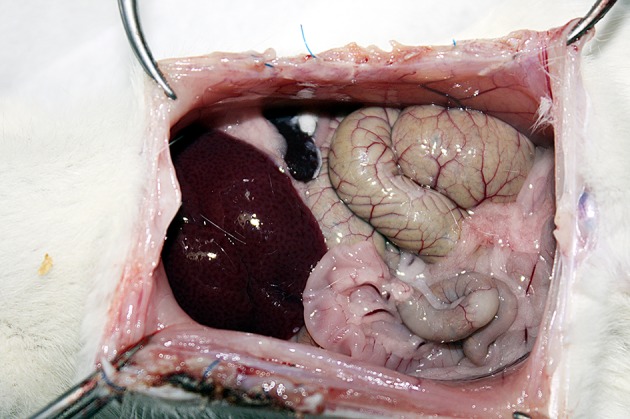
Postoperative View of Intraperitoneal Tacrolimus Administration

14 days after creation of the experiment model, all animals were euthanized with ether anesthesia, relaparotomy was performed to evaluate adhesion formation and rats’ peritoneal cavity were exposed through U-shaped incisions, providing maximal exposure (Figure 1).

The scoring system previously defined by Linsky et al. (12), was used for adhesion scoring. In this system, the involvement, resistance and severity of adhesion were evaluated separately, then the three values were added and the total adhesion score was calculated. Scoring was done according to the scale below.

Adhesion involvement scoring: no adhesion = 0, adhesion up to 25% of traumatized area = 1, adhesion up to 75% of traumatized area = 2, and adhesion up to 100% of traumatized area = 3. Resistance scoring: no adhesion=0, adhesions that can be separated without any resistance = 1, adhesions that can be separated with moderate power = 2, and adhesions that can be separated only with sharp dissection = 3. Severity scoring: no adhesion = 0, filmy and avascular = 1, moderately filmy and vascular = 2, dense and significantly vascular = 3. Three scores were summed for each rat and the total score was calculated, which is ranged between 0 and 12.

#### 3.2.2. Histopathological Evaluation

**Figure 2.**
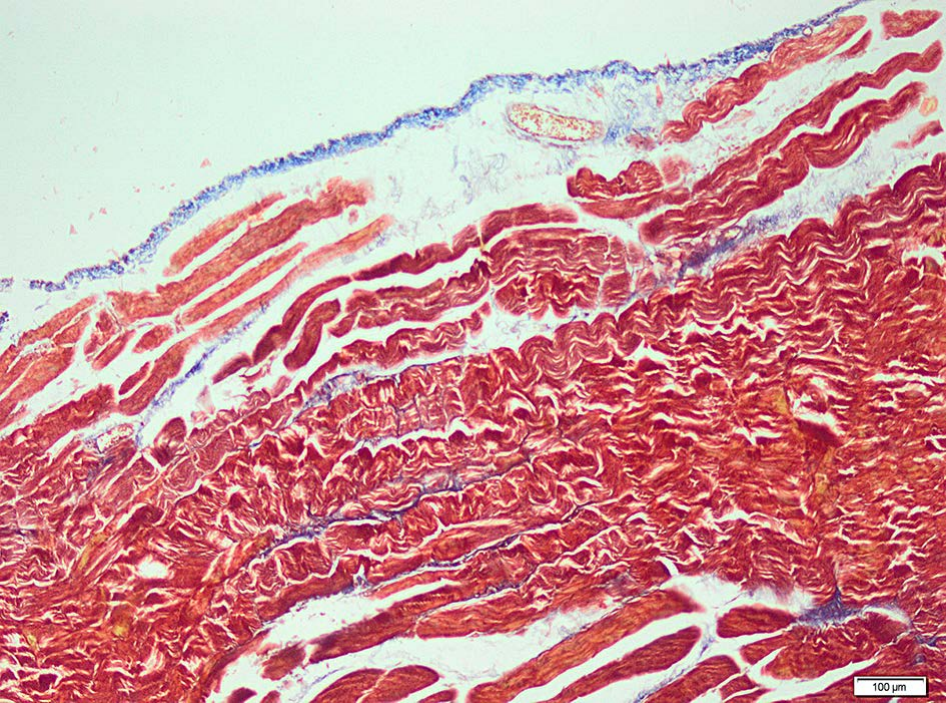
Microscopic View of Significant Fibrosis were Observed for the Control Group (MTx100)

**Figure 3.**
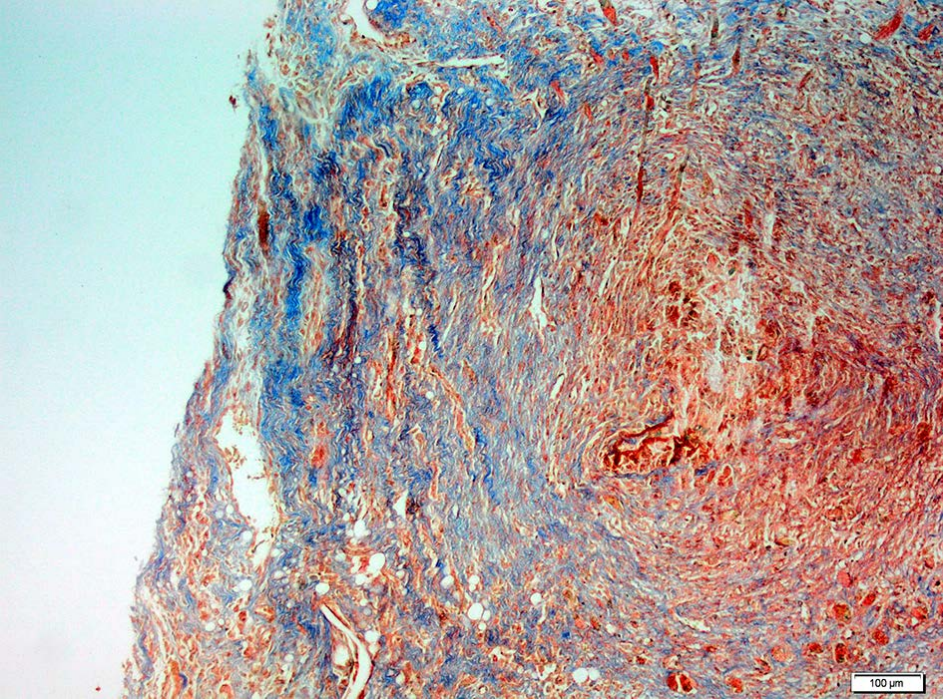
Microscopic View of Significant Fibrosis were Observed for the Control Group (MTx100)

Specimens were immediately collected after euthanization for histopathological examination. Histopathological examination was performed by light microscopy. The samples obtained from the abraded cecal tissue and the adjacent peritoneal tissues were ﬁxed in 10% neutral buffered formalin solution for 2 days. Tissues were washed in running water, and were dehydrated with ethanol. After dehydration, specimens were placed into xylene to obtain transparency and embedded in parafﬁn. Embedded tissues were cut in 5 μm-thick sections and were stained with hematoxylin/eosin and Masson’s trichrome for identification of fibrosis (Figure 2 and 3). 

Histopathological examinations were performed by a pathologist who was blinded to the study groups. The evaluated parameters were fibrosis, inflammation, and vascular proliferation, and each item was rated on a modified semi-quantitative scale of 0 - 3 (12). Histopathological evaluation was made according to the scale below. Fibrosis scoring: no fibrosis = 0; minimal, loose fibrosis=1; moderate fibrosis=2; and florid, dense fibrosis = 3. Inflammation scoring: no inflammation = 0; presence of giant cells, occasional lymphocytes and plasma cells = 1; presence of giant cells, plasma cells, eosinophils and neutrophils = 2; and presence of many inflammatory cells and microabscesses = 3. Vascular proliferation scoring: no vascular proliferation = 0; mild vascular proliferation = 1; moderate vascular proliferation=2; and intense vascular proliferation = 3.

#### 3.2.3. Statistical Analysis

Data were analyzed by using SPSS 15.00 software. Since measures related to the groups does not meet parametric test conditions, an alternative nonparametric Kruskal-Wallis H test was used in order to determine whether there are differences between the groups. In accordance with test results, Mann-Whitney U test was used in order to determine source of difference for significant differences. P < 0.05 value was accepted as significant. Monte Carlo Methods were also used for P value due to low sample size.

## 4. Results

Throughout the investigation, no animal died during or after surgery. A total of 98 rats sacrificed on postoperative 14th day.

**Table 1.** Kruskal-Wallis H test Results for Fibrosis, Inflammation, Vascular prolifeRation and Macroscopic Adhesion Measurements of the Experimental Groups and the Control Group.

	Mean ± SD.	Percentiles			Chi-Square	Monte Carlo P
		25.0	50 (Median)	75.0		
**Fibrosis (F)**					20.943	0.001 P < 0.01
G1	1.929 ± 0.616	2.0	2.0	2.0		
G2	1.143 ± 0.663	1.0	1.0	2.0		
G3	0.857 ± 0.864	0.0	1.0	2.0		
G4	1.000 ± 0.555	1.0	1.0	1.0		
G5	0.857 ± 0.363	1.0	1.0	1.0		
G6	1.286 ± 0.469	1.0	1.0	2.0		
G7	1.143 ± 0.864	0.0	1.0	2.0		
**Inflammation (I)**					9.674	0.131 P > 0.05
G1	1.143 ± 0.363	1.0	1.0	1.0		
G2	1.143 ± 0.663	1.0	1.0	2.0		
G3	1.000 ± 0.784	0.0	1.0	2.0		
G4	0.857 ± 0.663	0.0	1.0	1.0		
G5	0.857 ± 0.363	1.0	1.0	1.0		
G6	1.429 ± 0.514	1.0	1.0	2.0		
G7	1.000 ± 0.555	1.0	1.0	1.0		
**Vascular Proliferation (VP)**					20.010	0.001 P < 0.01
G1	1.714 ± 0.469	1.0	2.0	2.0		
G2	1.143 ± 0.663	1.0	1.0	2.0		
G3	0.714 ± 0.914	0.0	0.0	2.0		
G4	1.286 ± 0.726	1.0	1.0	2.0		
G5	0.571 ± 0.756	0.0	0.0	1.0		
G6	0.857 ± 0.663	0.0	1.0	1.0		
G7	1.000 ± 0.784	0.0	1.0	2.0		
**Macroscopic Adhesion (MA)**					12.963	0.040 P < 0.05
G1	4.857 ± 2.248	3.0	4.0	7.0		
G2	2.714 ± 1.437	2.0	3.0	3.0		
G3	2.000 ± 1.922	0.0	3.0	3.0		
G4	3.143 ± 1.512	3.0	3.0	4.0		
G5	2.571 ± 1.742	0.0	3.0	4.0		
G6	3.000 ± 1.359	3.0	3.0	4.0		
G7	2.571 ± 1.651	1.0	3.0	4.0		

Test results for fibrosis, inflammation, vascular proliferation and macroscopic adhesion measurements of the experimental groups and the control group.

Test results revealed that the difference between rank averages is significant for three out of four parameters whereas the difference for one parameter is not significant. The difference for groups is found to be significant for fibrosis levels (P = 0.001). Accordingly, the difference between rank average was also found out to be significant for vascular proliferation (P = 0.001). The differences between rank average was found out to be significant for macroscopic adhesion parameter (P = 0.040). However, the difference between rank average for inflammation as another parameter was not found out to be significant (P = 0.131). In comparison of adhesion grades, a significant difference was found between the control group and the experimental groups. However, there was not any significant difference between Groups 2 to 7 (Table 2). 

**Table 2.** Paired Statistical Comparisons for Fibrosis (F), Vascular Proliferation (VP) and Macroscopic Adhesion (MA) Between the Control Group and the Experimental Groups.

Groups	P value for F	P value for VP	P value for MA
**G1-G2**	0.008	0.031	0.008
**G1-G3**	0.002	0.004	0.002
**G1-G4**	0.001	0.139	0.124
**G1-G5**	0.000	0.000	0.043
**G1-G6**	0.012	0.002	0.089
**G1-G7**	0.022	0.015	0.012

In all of experimental groups, decrease in fibrosis was statistically significant. Fibrosis was decreased most prominently in intraperitoneal tacrolimus group (P = 0.000), and decrease was least in intraperitoneal cyclosporine group (P = 0.022). Vascular proliferation was significantly decreased in all experimental groups (P < 0.05) except for systemic tacrolimus group (P = 0.139). Most prominent reduction in vascular proliferation was for intraperitoneal tacrolimus group (P = 0.000). Decrease in macroscopic adhesion scores were statistically significant in intraperitoneal mycophenolate mofetil (P = 0.002), systemic mycophenolate mofetil (P = 0.008) and intraperitoneal cyclosporine (P = 0.012) groups. There was not statistically significant decrease in remaining experimental groups.

## 5. Discussion

Postsurgical adhesion formation is a significant clinical problem for any of visceral surgery and the development of preventive strategies against adhesion formation has become the major goal of numerous investigations. Several drugs and substances had been historically used locally or systematically for this purpose, including mechanical barriers and physical, chemical, and pharmacological agents (13, 14). However, despite promising reports, none of these have been approved as a standard therapy (15).

Former experimental studies on peritoneal adhesion proposed a vital role for inflammation in adhesion formation. In a surgically-induced cecal abrasion model in mice, Th1 CD4 αβ cells are shown to be critical in peritoneal adhesion formation, and shortly after tissue injury and throughout adhesiogenesis these activated T-cells become the predominant cells in the peritoneal cavity (16). Several chymokines and cytokines, including interleukin-1, M-CSF, GM-CSF, and MCP-1 have also regulatory roles in fibrinolytic process (17, 18). In this study, we created a cecal abrasion model in rats and investigated the influence of immunosuppression on the development of adhesions. Immunosuppressive drugs have various grades of immunoregulatory influences on a wide range of intra- and intercellular actions from antigen defining to cell proliferation and cytokine production (19).

Although there are no structural similarities between cyclosporine-A (CsA) and tacrolimus (FK506) and their biological activities are different, with connection of cyclosporine-A/cyclophilin and tacrolimus/FK-binding protein (FKBP) to target molecule Calcineurin, a dephosphorylation occurs on target molecule in the cell and this results in inhibition of IL-2 gene and the other cytokine-related genes. Therefore, production of IL-2 is blocked and proliferation signs of T-cells are inhibited (20, 21). Mycophenolate mofetil is a kind of pro-drug of mycophenolic acid (MPA) and fermentation products of Penicillium types (22). Mycophenolic acid is a potent inhibitor of inosine-monophosphate dehydrogenase, which is an important enzyme for de novo purine synthesis (23). Consequently, along with other cells, mycophenolic acid has a potent inhibitory role on lymphocytes. Thereby, MPA suppresses cell-mediated immune response and antibody formation (24, 25).

Several immunosuppresive agents have been shown to reduce postoperative intraperitoneal adhesions in experimental models. Formerly, tacrolimus has reduced peritoneal adhesions after a bowel transplantation model in rats (24). Another immunosuppressive drug sirolimus, have been found to reduce intraabdominal adhesions in an intraabdominal prosthetic vascular graft model and ventral prosthetic mesh model (26, 27).

In this study we aimed to investigate the effect of immunosuppressive drugs on adhesion formation and to compare most commonly used immunosuppressive drugs. In order to understand whether effect of drug is local or systemic, we constituted topical and systemic administered drug groups. Doses were adjusted according to bioavailability of drugs so as to provide similar systemic drug concentrations between topically and systemically administered drugs. We chose to study on most commonly used immunosuppressive agents with well- known side effect profiles.

We have found that immunosuppressive drugs have significant effect on histopathological findings of adhesion. Also there were significantly less adhesion in intraperitoneal mycophenolate mofetil, systemic mycophenolate mofetil and intraperitoneal cyclosporine groups.

Immunosuppressive drugs have well-known adverse effects of wound healing impairment. In our study, there were no evidence of wound healing problem in any of rat, nor was any adverse effect that can be attributed to immunosuppressive medication encountered. Study has exhibited that topical use of immunosuppressive agents prevents postoperative peritoneal adhesions without systemic effects of immunosuppressive medication. Topical use of these medications may provide novel approaches for postoperative adhesion prevention. On the other hand, although drug doses are determined according to ordinary doses used for immunosuppression, these are eventually empirical doses. Clinical use of immunosupressants for adhesion prevention requires further studies along with this experimental animal study.

In conclusion, immunosuppressive drugs seem to reduce the intraperitoneal adhesions significantly without any prominent adverse effect or any compromise on wound healing in therapeutic dose range. These drugs may promise an effective solution for postoperative intraperitoneal adhesions and its ominous complications.

## References

[1] Restrepo C, Chacon J, Manjarres G (2010). Fungal peritonitis in peritoneal dialysis patients: successful prophylaxis with fluconazole, as demonstrated by prospective randomized control trial.. Perit Dial Int..

[2] Du X, Hong G, Sun P, Liu G (2012). Zn2+-SCMC versus HA for preventing intraperitoneal adhesions: a rat model study.. Int J Med Sci..

[3] Kossi JA, Salminen PT, Laato MK (2004). Surgical workload and cost of postoperative adhesion-related intestinal obstruction: importance of previous surgery.. World J Surg..

[4] Burnett SH, Beus BJ, Avdiushko R, Qualls J, Kaplan AM, Cohen DA (2006). Development of peritoneal adhesions in macrophage depleted mice.. J Surg Res..

[5] Liakakos T, Thomakos N, Fine PM, Dervenis C, Young RL (2001). Peritoneal adhesions: etiology, pathophysiology, and clinical significance. Recent advances in prevention and management.. Dig Surg..

[6] Cheong YC, Laird SM, Li TC, Shelton JB, Ledger WL, Cooke ID (2001). Peritoneal healing and adhesion formation/reformation.. Hum Reprod Update..

[7] Sulaiman H, Dawson L, Laurent GJ, Bellingan GJ, Herrick SE (2002). Role of plasminogen activators in peritoneal adhesion formation.. Biochem Soc Trans..

[8] Yang B, Gong C, Zhao X, Zhou S, Li Z, Qi X (2012). Preventing postoperative abdominal adhesions in a rat model with PEG-PCL-PEG hydrogel.. Int J Nanomedicine..

[9] Kato T, Ruiz P, Thompson JF, Eskind LB, Weppler D, Khan FA (2002). Intestinal and multivisceral transplantation.. World J Surg..

[10] Sugitani A, Gritsch HA, Shapiro R, Bonham CA, Egidi MF, Corry RJ (1998). Surgical Complications in 123 Consecutive Pancreas Transplant Recipients: Comparison of Bladder and Enteric Drainage.. Transplantation proceedings..

[11] Blachar A, Federle MP (2001). Bowel obstruction following liver transplantation: clinical and ct findings in 48 cases with emphasis on internal hernia.. Radiology..

[12] Irkorucu O, Ferahkose Z, Memis L, Ekinci O, Akin M (2009). Reduction of postsurgical adhesions in a rat model: a comparative study.. Clinics (Sao Paulo)..

[13] Kaptanoglu L, Kucuk HF, Yegenoglu A, Uzun H, Eser M, Mentes CV (2008). Effects of seprafilm and heparin in combination on intra-abdominal adhesions.. Eur Surg Res..

[14] Kesting MR, Loeffelbein DJ, Steinstraesser L, Muecke T, Demtroeder C, Sommerer F (2008). Cryopreserved human amniotic membrane for soft tissue repair in rats.. Ann Plast Surg..

[15] Hellebrekers BW, Trimbos-Kemper GC, van Blitterswijk CA, Bakkum EA, Trimbos JB (2000). Effects of five different barrier materials on postsurgical adhesion formation in the rat.. Hum Reprod..

[16] Chung DR, Chitnis T, Panzo RJ, Kasper DL, Sayegh MH, Tzianabos AO (2002). CD4+ T cells regulate surgical and postinfectious adhesion formation.. J Exp Med..

[17] Chegini N (2002). Peritoneal molecular environment, adhesion formation and clinical implication.. Front Biosci..

[18] Falk K, Bjorquist P, Stromqvist M, Holmdahl L (2001). Reduction of experimental adhesion formation by inhibition of plasminogen activator inhibitor type 1.. Br J Surg..

[19] Kansu E (2002). Immünosüpressif Ajanların Genel Özellikleri ve Etki mekanizmaları.. Ankem Dergisi ..

[20] Horowitz MM, Przepiorka D, Bartels P, Buell DN, Zhang MJ, Fitzsimmons WE (1999). Tacrolimus vs. cyclosporine immunosuppression: results in advanced-stage disease compared with historical controls treated exclusively with cyclosporine.. Biol Blood Marrow Transplant..

[21] Nakajima K, Ochiai T, Nagata M, Suzuki T, Gunji Y, Asano T (1993). Effects of triple therapy of cyclosporine, FK 506, and RS-61443 on allogeneic small bowel transplantation in dogs.. Transplant Proc..

[22] McMurray RW, Harisdangkul V (2002). Mycophenolate mofetil: selective T cell inhibition.. Am J Med Sci..

[23] Allison Anthony C, Eugui Elsie M (2000). Mycophenolate mofetil and its mechanisms of action.. Immunopharmacology..

[24] Wasserberg N, Nunoo-Mensah JW, Ruiz P, Tzakis AG (2007). The effect of immunosuppression on peritoneal adhesions formation after small bowel transplantation in rats.. J Surg Res..

[25] Allison AC (2005). Mechanisms of action of mycophenolate mofetil.. Lupus..

[26] Kanko M, Ozbudak E, Ozerdem A, Aksoy A, Kilic M, Berki KT (2006). Effect of sirolimus in the prevention of adhesions around intraabdominal prosthetic graft.. World J Surg..

[27] Maciver AH, McCall MD, Edgar RL, Thiesen AL, Bigam DL, Churchill TA (2011). Sirolimus drug-eluting, hydrogel-impregnated polypropylene mesh reduces intra-abdominal adhesion formation in a mouse model.. Surgery..

